# Patterns of Gastrointestinal Helminth Infections in *Rattus rattus, Rattus norvegicus*, and *Mus musculus* in Chile

**DOI:** 10.3389/fvets.2022.929208

**Published:** 2022-06-28

**Authors:** Alexandra Grandón-Ojeda, Lucila Moreno, Carolina Garcés-Tapia, Fernanda Figueroa-Sandoval, Jazmín Beltrán-Venegas, Josselyn Serrano-Reyes, Bárbara Bustamante-Garrido, Felipe Lobos-Chávez, Hellen Espinoza-Rojas, María Carolina Silva-de la Fuente, AnaLía Henríquez, Carlos Landaeta-Aqueveque

**Affiliations:** ^1^School of Biological Sciences, University of Bristol, Bristol, United Kingdom; ^2^Departamento de Zoología, Facultad de Ciencias Naturales y Oceanográficas, Universidad de Concepción, Concepción, Chile; ^3^Departamento de Patología y Medicina Preventiva, Facultad de Ciencias Veterinarias, Universidad de Concepción, Chillán, Chile; ^4^Departamento de Ciencias Agrarias, Facultad de Ciencias Agrarias y Forestales, Universidad Católica del Maule, Los Niches, Chile; ^5^Facultad de Medicina Veterinaria, Universidad San Sebastián, Concepción, Chile

**Keywords:** coinfection, helminthiasis, invasive rodents, mice, rats, sex-biased parasitism, spillback, rodent diseases

## Abstract

Few studies have assessed the patterns of parasite populations of rodents over a longitudinal gradient in Chile. In this work, the gastrointestinal helminthic fauna of invasive rodents in Chile was examined to assess the association between their presence/absence and abundance with latitude, host sex, and host body condition, and to assess the coexistence and correlation of the abundance between parasite species. Rodents were obtained from 20 localities between 33 and 43°S. Helminths were extracted from the gastrointestinal tract and identified morphologically. Overall, 13 helminth taxa were obtained. The most frequently identified parasite species was *Heterakis spumosa*, and the most abundant was *Syphacia muris*, while *Physaloptera* sp. was the most widely distributed. No locality presented with a coexistence that was different from that expected by chance, while the abundance of five helminthic species correlated with the abundance of another in at least one locality, most likely due to co-infection rather than interaction. Host sex was associated with parasite presence or abundance, and female sex-biased parasitism was notably observed in all cases. Body condition and latitude presented either a positive or negative association with the presence or abundance of parasites depending on the species. It is notable that the likely native *Physaloptera* sp. is widely distributed among invasive rodents. Further, gravid females were found, suggesting spillback of this species to the native fauna. The low frequency and abundance of highly zoonotic hymenolepid species suggest that rodents are of low concern regarding gastrointestinal zoonotic helminths.

## Introduction

Invasive rodents, mainly *Mus musculus, Rattus rattus*, and *Rattus norvegicus* with a worldwide distribution, and *Rattus exulans* with a Pacific distribution, have been recognized as they perform several roles ecologically (1) and epidemiologically (2). From an ecological standpoint, invasive rodents not only directly impact native communities by predation or competition (3), but they also indirectly impact communities by both introducing allochthones parasites or amplifying native ones (4). From an epidemiological and public health standpoint, rodents are important reservoir hosts of several zoonotic helminths (5–9), and, although several rodents can harbor zoonotic parasites (10–12), most of the reported zoonotic parasites have been found in invasive rodents (*Rattus* spp. and *Mus* spp.) (13).

In Chile, some studies have assessed the parasitism of rodents by helminths at the parasite level, at the host population level, and the host community level. Thus, respectively, those studies have described new species (14, 15), component populations, and communities (16, 17), and they have assessed the sharing of parasites among different host populations within a host community (18, 19); however, few have focused merely on the parasites of an invasive rodent species. Existing research has explored parasites in reduced geographical areas (20–22), while another included invasive rodents in a host community-level study (19). Studies focused on zoonotic helminths in invasive rodents mostly focused on a single parasite species, *Trichinella spiralis* (23, 24), even though many zoonotic helminths that were reported in rodents in Chile are parasites introduced with invasive rodents (13).

Studies focused on the ecology of helminths in these invasive rodents have assessed the sharing of helminths with native rodents (19). Little information is available about what happens within component communities, and no studies have examined the factors affecting the presence and abundance of these parasites. It is known that the coexistence of parasite infrapopulations could lead to either increased loads (synergism) or reduced loads (antagonism), given that different causes lead to direct interaction or immune-mediated interactions (25). Although the coexistence or lack of coexistence can be caused by interactions, they can be also caused by similar or different infection routes, respectively, among other factors (26), and their study is worthy. Thus, the objective of this study was to describe the gastrointestinal helminthic fauna of introduced rodents in Chile along a latitudinal gradient, to assess the association between the occurrence (presence/absence) of parasites and their abundance with latitude, and to host sex and host body condition. Further, this study also assessed the coexistence and association between the abundance of parasite species.

## Materials and Methods

**Figure 1:**
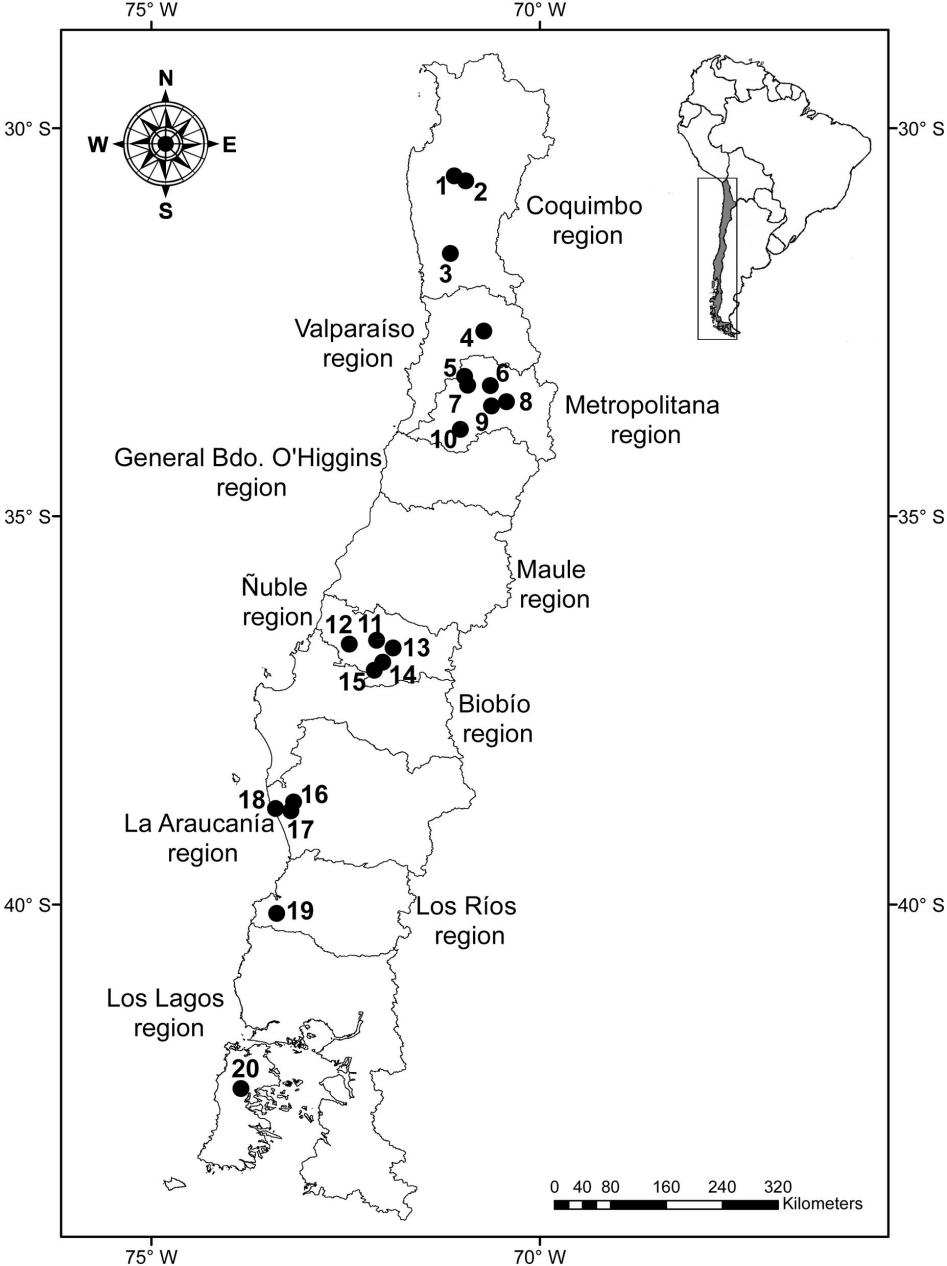
Map of Chile with the localities included in the study: 1 = Sotaquí, 2 = Monte Patria, 3 = Illapel, 4 = Putaendo, 5 = Maipú 1, 6 = San Ramón, 7 = Maipú 2, 8 = La Pintana, 9 = Calera de Tango, 10 = Talagante, 11 = Chillán, 12 = Nueva Aldea, 13 = Pinto, 14 = El Carmen, 15 = Pemuco, 16 = Carahue, 17 = Collico, 18 = Puerto Saavedra, 19 = Alerce Costero National Park, and 20 = Castro.

The *Rattus rattus* (*n* = 159), *R. norvegicus* (*n* = 30), and *Mus musculus* (*n* = 91) included in this study were obtained from previous studies, which had other objectives (19, 24, 27, 28); the trapping and euthanasia by an overdose of anesthesia of those rodents were described in those articles. In addition to ethic and/or legal authorizations provided for these articles, new samplings were approved by the Comité de Bioética of the Facultad de Ciencias Veterinarias (CBE-34-2019) of the Universidad de Concepción. Several localities were included in the study (Table 1), most of which were visited once; however, some localities were visited two or three times. Therefore, we defined a study unit (SU) as a set of host specimens obtained from a single locality over <31 days. Thus, two SUs from the same locality were not expected to represent the same parasite community given the expected temporal variation of the presence and abundance of parasites (29). A total of 27 SUs were studied between the Coquimbo and Los Lagos regions (see the localities in Figure 1). The gastrointestinal tract was examined for the presence of helminths under a stereomicroscope. Nematodes were cleared with lactophenol or ethanol–glycerin, and cestodes were stained with carmine–HCl, and they were identified under a light microscope following the keys of Anderson et al. (30) and Khalil et al. (31).

**Table 1:** Abundances of helminths in allochthonous rodents in Chile by host species and study unit.

SU^*^	**Locality**	Host species^*^	**Sample size**	H.s.^*^	S.spp.^*^, ^***^	A.t.^*^	T.m.^*^	N.b.^*^	Ph.sp.^*^	Pt.sp.^*^	Hy^*^, ^****^	Pr.sp.^*^	G.n.^*^	Ca.^*^
				MA^*^, ^**^	**MA**	**MA**	**MA**	**MA**	**MA**	**MA**	**MA**	**MA**	**MA**	**MA**
1	Sotaquí	R. r.	1	0	0	0	0	0	0	0	0	0	0	0
2	Sotaquí	R. r.	1	0	*n* = 1	0	0	0	*n* = 1	0	0	0	0	0
3	Monte Patria	R. r.	3	0	*n* = 2	0	0	0	*n* = 1	0	0	*n* = 1	0	0
4	Illapel	R. r.	6	0	0	0	0	0	0	0	0	0	0	0
5	Illapel	R. r.	5	*n* = 5	0	0	0	0	*n* = 1	0	*n* = 3	0	*n* = 1	0
6	Putaendo	R. n.	3	0	*n* = 3	0	0	0	0	0	*n* = 1	0	0	0
6	Putaendo	R. r.	3	0	*n* = 8	0	0	0	0	0	0	0	0	0
7	Maipú 1	R. r.	1	0	*n* = 106	0	0	0	0	0	0	0	0	0
7	Maipú 1	M. m.	1	0	*n* = 15	0	0	0	0	0	0	0	0	0
8	Maipú 1	R. n.	1	0	*n* = 1,358	0	0	0	0	0	0	0	0	0
8	Maipú 1	R. r.	1	0	0	0	0	0	*n* = 5	0	0	0	0	0
9	San Ramón	R. r.	5	0	0	*n* = 11	0	0	0	0	0	0	0	0
10	Maipú 2	R. r.	3	*n* = 3	*n* = 43	0	0	*n* = 1	*n* = 7	0	*n* = 1	0	0	0
10	Maipú 2	R. n.	12	25.8	3.42	0	0	2.08	0.58	0.25	0.33	0	0	18.3
10	Maipú 2	M. m.	13	0	5.85	0	0	0	0.08	0	0	0	0	0
11	Maipú 2	R. r.	3	*n* = 2	0	0	0	0	*n* = 7	0	0	0	0	0
11	Maipú 2	M. m.	16	0	74.8	0	0	0	1.9	0	0	0	0	0
12	La Pintana	R. n.	6	0	*n* = 44	0	0	0	0	0	*n* = 2	0	0	0
12	La Pintana	M. m.	27	0	2.07	0.07	0	0	0.04	0	0	0	0	0
13	La Pintana	R. r.	1	0	0	0	0	0	0	0	0	0	0	0
13	La Pintana	M. m.	9	0	*n* = 358	*n* = 2	0	0	*n* = 1	0	0	0	0	0
14	La Pintana	M. m.	18	0.78	27.8	0	6.78	0	0	0	0	0	0	0
14	La Pintana	R. r.	1	0	0	0	0	0	0	0	0	0	0	0
15	Calera de tango	R. n.	1	0	0	0	0	0	0	0	0	0	0	0
16	Talagante	R. n.	1	0	0	0	0	0	0	0	0	0	0	0
17	Talagante	R. n.	1	*n* = 1	0	0	0	0	0	0	0	0	0	0
17	Talagante	R. r.	8	0	*n* = 431	*n* = 1	*n* = 1	0	0	0	0	0	0	0
18	Chillán	*R. r*.	16	0	0	0	0	0	0	0	0	0	0	0
19	Nueva Aldea	*R. r*.	8	*n* = 20	*n* = 1	0	0	0	*n* = 8	*n* = 1	0	*n* = 3	0	*n* = 1
20	Pinto	*R. r*.	14	7.43	0	0	0	26.3	0.571	0.36	1.14	2.2	0	0
20	Pinto	*R. n*.	1	0	0	0	0	0	0	0	0	0	0	0
21	El Carmen	*R. r*.	6	*n* = 1	0	0	0	0	0	0	0	0	0	0
22	Pemuco	*R. r*.	20	0	0	0	0	0	0	0	0	0	0	0
22	Pemuco	*R. n*.	3	0	0	0	0	0	0	0	0	0	0	0
23	Carahue	*M. m*.	7	0	0	0	0	0	0	0	0	0	0	0
23	Carahue	*R. r*.	21	2.1	84.4	0	0	3.29	0.14	0	0	0	0	0
24	Collico	*R. r*.	6	*n* = 20	*n* = 146	0	0	*n* = 154	*n* = 2	0	0	0	0	0
25	Puerto Saavedra	R. r.	2	*n* = 32	*n* = 109	0	0	*n* = 13	*n* = 5	0	0	0	0	0
26	Alerce Costero National Reserve	*R. r*.	10	0	0	0	0	0	0.2	0.3	0	0	0	0
27	Castro	*R. r*.	14	3.3	5.36	0	0	0	0	0	0	0	0	0
27	Castro	*R. n*.	1	*n* = 3	0	0	0	0	0	0	0	0	0	0

* *SU, Study Unit; MA, mean abundance; R. r., Rattus rattus; R. n., Rattus norvegicus; M. m., Mus musculus; H.s., Heterkis spumosa; S., Syphacia; A.t., Aspiculuris tetraptera; T.m., Trichuris muris; N.b., Nippostrongylus brasiliensis; Ph, Physaloptera; Pt., Pteryogodermatites; Hy., Hymenolepididae; Pr., Protospirura; G.n., Gongylonema neoplasticum; Ca., Capillariidae*.

** *In small sample size SU n = X indicates the abundance instead of MA*.

*** *Syphacia spp. are S. obvelata in M. m. and S. muris in Rattus sp*.

**** *SU 5: one parasite specimen is the sole Rodentolepis nana found in the study. The rest were Hymenolepis diminuta*.

The prevalence (95% confidence interval [CI] with the Clopper–Pearson method), mean abundance (95% CI estimated by the bootstrapping method) (32), variance-to-mean range (V-M), Poulin's discrepancy index (D), and *k* parameter from a negative binomial distribution (33) were estimated by parasite population within the host species and SU. Species richness was estimated with the Chao2 method (34) by SU when the presence of frequent and rare species enabled the estimation of confidence intervals. Confidence intervals were obtained using the Quantitative Parasitology online application, QPweb (34).

Patterns of species co-occurrence at the component community, defining it at the SU level, were also examined. For this, we used the EcoSim version 7.0 (35) software to test for non-random patterns of species co-occurrence for each locality with the C-score index (36). We selected SUs that had at least 12 hosts with at least three parasite species with two of them presenting prevalence higher than 20%. The C-score measures the average number of “checkerboard units,” i.e., no co-occurrence, among all possible species pairs. We used the fixed–fixed model with 5,000 iterations. When the observed C-score index is higher than the simulated C-score, it indicates that the helminth species co-occur less frequently than by chance. Conversely, an observed C-score lower than the simulated score suggests that the helminth species co-occur more frequently than expected by chance. On the other hand, if the observed C-scores do not differ statistically from the simulated C-scores, then random patterns of species co-occurrence are observed (37). In addition, given that the abundance of parasites reflects the number of infection events, two species that co-occur are expected to have correlated abundance; therefore, we also tested whether the abundance of a species was associated with the abundance of the other species, between pairs of parasite species within SUs with more than 10 hosts, using Spearman correlation tests.

Finally, we assessed the association between the occurrence (presence/absence) and abundance of parasite species with the latitude, body condition, and sex of the host using multifactorial logistic and binomial negative regressions, respectively. In the case of the variable sex, “female” was considered as a basal level and “male” as a dummy variable, latitude was measured as degree and decimals, and body condition was calculated with Fulton's index (K = mass/lenght^3^). To select the best model, we began with the full model containing the three independent variables. The *P*-value of the variables was only considered as a criterion that was used to select the variable to remove in each step, with the variable with the highest *P*-value being the variable to remove. Likelihood ratio tests (LR tests) were used to choose the best model, where the removal of any variable implied a significant loss of likelihood and took place with a model *P*-value of ≤ 0.05. In addition, in the case of logistic regressions, goodness-of-fit (GOF) tests were performed to assess the selected models. If the observed parasite presence did not fit the expected model, the variable was not considered to be associated with the occurrence. Regressions, LR tests, GOF tests and, correlation tests were performed using Stata/BE 17 (StataCorp LLC). The significance level of the models and tests was *P* = 0.05. Only significant associations are reported in the results.

## Results

Overall, the observed species richness was 13 species, and the estimated richness was 14 (95% CI: 14.1–27.1). The taxa found herein were: *Heterakis spumosa, Syphacia obvelata, Syphacia muris, Aspiculuris tetraptera, Trichuris muris, Nippostrongylus brasiliensis, Physaloptera* sp., *Protospirura* sp., *Pterygodermatites* (*Paucipectines*) sp., *Gongylonema neoplasticum*, Capillariidae, *Hymenolepis diminuta*, and *Rodentolepis nana*. The most frequently found parasite among examined animals was *H. spumosa* with 25.4% (CI 19.4–32.2. Mean abundance: 3.03; CI 1.91–5.13), identified among *Rattus* spp. hosts and was found in 12 SUs. *Syphacia muris* was the second most frequently found with a 17.5% (CI 12.3–23.6) prevalence rate among *Rattus* spp., but it presented the highest mean abundance among *Rattus* sp. (21.9; 9.6–50.6) and was present in 14 SUs. *Physaloptera* sp., with an overall 9.6% prevalence rate and 0.225 mean abundance, was the most widely distributed species, present in 17 SUs, including the northernmost and southernmost studied localities. Conversely, the rarest taxa were *A. tetraptera* and Capillariidae, which were found in only four individuals each. When considering parasites with an aggregation index measured in more than one SU, *S. muris* presented the highest average V-M = 197, with a maximum of 520 in a single SU, followed by *H. spumosa* which presented an average V-M = 14.6 with a maximum of 33.9. Conversely, *Physaloptera* sp. presented the highest average D-index, 0.85 (maximum 0.93), followed by *S. muris*, 0.82 (max. 0.85). *Syphacia obvelata* presented the lowest *k* parameter, 0.11, followed by *Physaloptera* sp., 0.21; however, this parameter was not obtained in many samples given that the maximum likelihood estimate of *k* could not be computed, which may be due to a lack of fitness with the negative binomial distribution given the small local sample size or its very low local prevalence. The abundances by host species and SU are given in Table 1. The details of the prevalence, mean abundance, and aggregation indices by host species and SU, as well as the estimated richness by SU, are given in Supplementary Table 1.

We analyzed the co-occurrence between parasite species in three-component communities, which showed a C-score similar to that expected by chance, indicating that the parasite species co-occur as frequently as expected (Supplementary Table 2).

On the other hand, among 45 pairwise correlation tests of abundance between parasite populations, only five significant associations were found, all of which were positive: *H. spumosa*–*S. muris* (ρ = 0.45; *P* = 0.04), *H. spumosa*–*N. brasiliensis* (ρ = 0.55; *P* = 0.01) and *S. muris*–*N. brasiliensis* (ρ = 0.6; *P* < 0.01) in Carahue; and *H. spumosa*–*N. brasiliensis* (ρ = 0.63; *P* = 0.02) and *Physaloptera* sp.–*Protospirura* sp. (ρ = 0.73; *P* < 0.01) in Pinto. The details of all pairwise tests with their ρ and *P*-values are given in Supplementary Table 3.

Finally, sex was associated with the presence of *H. spumosa* [odds ratio (OR) = 0.37], *N. brasiliensis* (OR = 0.16), and *H. diminuta* (OR =0.67) and was associated with the abundance of *T. muris* (coefficient = −3.88), *N. brasiliensis* (coef. = −5.18), and *Physaloptera* sp. (coef. = −1.36); the presence was more frequent and/or the abundance was higher in females than in males in all cases. Body condition was negatively associated with the occurrence of *H. spumosa* (OR = 0.67) and *Physaloptera* sp. (OR = 0.41), negatively associated with the abundance of *H. spumosa* (coef. = −1.2) and positively associated with the abundance of *S. obvelata* (coef. = 1.83). Latitude was positively associated with the occurrence (OR = 1.21) and abundance (coef. = 0.47) of *H. spumosa*, and negatively associated with the occurrence of *H. diminuta* (OR = 0.67) and the abundance of *Physaloptera* sp. (coef. = −0.32) and Capillariidae (coef. = −1.62). The details of the selected models are given in Supplementary Tables 4–13. When the *P*-value of the variable was higher than 0.05, the LR-test output that the remotion of the variable caused a significant loss of likelihood. In each selected model, the likelihood of the model was significantly higher than the null model (see the *P*-value of the log-likelihood).

## Discussion

A total of 13 gastrointestinal helminth species were found; however, the richness estimation suggests that additional work is necessary to better determine parasite richness and to identify all parasite species inhabiting the gastrointestinal tracts of rats and mice in Chile. Two of the helminth species reported herein, *H. diminuta* and *R. nana*, have been frequently reported to infect humans elsewhere (38–40); however, they were found with low frequency and abundance in the present study. Other zoonotic species, *S. obvelata*, and *S. muris*, were more prevalent and abundant in their hosts, *M. musculus* and *Rattus* spp., respectively, but there are only a few reports of them infecting humans (41, 42). Finally, *T. muris* has also been seldomly reported to infect humans, and was scarcely found in this study. Thus, the results suggest that, regarding helminthic infections, invasive rodents in Chile are of a minor, but not null, concern from a public health standpoint since their cycles are maintained by these invasive rodents. This aligns with the lack of reports of *Syphacia* infection in Chilean people coupled with the decreasing prevalence of hymenolepids (43).

In addition to the five mentioned helminthic species, *H. spumosa, A. tetraptera, N. brasiliensis*, and *G. neoplasticum* have been reportedly found in *Rattus* spp. and *M. musculus* elsewhere in both laboratory and feral specimens (8, 42, 44–47). The enemy release hypothesis states that invasive animals present with fewer parasite species than in their original territory (48) given the small sample size of translocated animals, the loss of parasites during the translocation process, and/or the adaptation of the parasite to the new territory (49). Thus, the number of parasite species reported herein is larger than expected since it is similar to the richness reported in the Palearctic (50, 51), but higher than in other invaded territories (52). This suggests that there have been several introduction processes, i.e., processes of translocation of rodents from overseas to Chile, that favor the sampling of different parasite species in each introduction process. The low prevalence and abundance of many parasite species may favor the process of invasion by these allochthones rodents. Conversely, some parasites reported herein have not been reported in *Rattus* spp. and *Mus musculus* with worldwide distribution; rather, they seem to be native parasites from Neotropical rodents. *Physaloptera* sp. and *Protospirura* sp. are parasite genera frequently reported in native rodents in Chile (16, 19, 53, 54), and in both cases, gravid females were found, which supports the hypothesis that there is a spillback of parasites (55–57); however, new temporal studies are necessary to determine whether the parasitic loads increase in native rodent populations after the arrival of invasive rodents. In the case of *Pterygodermatites* sp., although the species was not identified given the low availability of males worms, species found in native rodents in Chile (19) are morphologically different from that reported herein, at least in terms of the distance of the first cuticular projection and the anterior end, as well as and the number of cuticular projections, suggesting that it could be a co-introduced species. *Pterygodermatites* spp. have been reported previously in *Rattus* spp. in Taiwan (58) and Thailand (46), with *P. tani* and *P. whartoni* being the reported species.

The positive associations between parasites agree with the fact that they have similar cycles. Thus, *S. muris, H. spumosa*, and *N. brasiliensis*, which co-abound in Carahue, have a direct cycle, suggesting that the correlation of their abundance could be due to similar transmission methods. On the other hand, *Physaloptera* sp. and *Protospirura* sp. have an indirect cycle, with Orthoptera, Coleoptera, and Dictyoptera insects being reported as intermediates hosts of *Physaloptera* sp. (59), and Dermaptera insects being reported as intermediate hosts of *Protospirura* sp. (60). In addition, both helminthic taxa seem to have native rodents as part of their reservoir, insofar as their coexistence with native rodents and their predatory behavior on insects are factors that favor the infection of both parasites by the same host. Therefore, although correlated abundances do not seem to be the general rule, which agrees with the lack of significant co-occurrence, the results suggest that if they do exist, they can result from co-infection rather than interspecies interactions between parasite species.

Sex is reportedly a significant factor affecting the presence and abundance of parasites; however, female sex-biased parasitism is rare in the literature (61), as males are usually more likely to be parasitized than females (29, 62–64). Some factors, such as pregnancy and lactation, have been mentioned in the literature as weakening the resistance of female mammals to parasitic infection (65–67); however, in this study, the pregnancy or lactation conditions of female rodents were not recorded transversally, and this factor could not be assessed. Behavioral factors, such as feeding, have also been assessed, with female bank voles more likely to be exposed to spirurid parasites given their higher proportion of invertebrate animal consumption (61, 68). However, this differential behavior should be assessed in invasive rats, and might only explain the association between sex, the presence of *H. diminuta*, and the abundance of *Physaloptera* sp., which are transmitted by invertebrate intermediate hosts.

There were no common patterns of variation related to the body condition-associated parasitism, as most cases were not significantly associated. Significant associations may have been because the larger host's body could offer more resources to the parasite (69), that larger hosts could have offered greater opportunities for parasite infection (70) (positive association) or that parasitism could result in damage to the host (negative association) (70, 71). It is not easy to explain that the body condition is positively associated with the infection with *S. obvelata* and negatively associated with the infection with *H. spumosa*, given that both parasites present a direct cycle. Thus, results suggest that *H. spumosa* is more pathogenic than *S. obvelata*. However, this difference could be influenced by the host, since *S. obvelata* is mainly a parasite of *M. musculus* while *H. spumosa* parasitize mainly *Rattus* spp. Further studies are necessary to test these hypotheses. On the other hand, the negative association of *Physaloptera* sp. infection with the body condition suggests a damage to the host by the parasite, which is in agreement with previous records in other species (72, 73).

*Heterakis spumosa* was more prevalent and abundant in the south, which suggests that cold and humid climates favor the persistence of infecting stages of this parasite in the environment. Conversely, *H. diminuta* and *Physaloptera* sp. were more prevalent or abundant to the north, which suggests that warmer or drier climates favor their intermediates hosts. Capillariidae was also more abundant to the north, but further studies are needed to determine the species and the life cycle of this taxa to understand this association. The contrasting associations between latitude and parasitic rates suggest that the persistence of free-living parasites, or the intermediate host stages of parasites, could explain these associations. More studies assessing these hypotheses are necessary to establish a cause.

## Conclusion

In this work, we reported 13 gastrointestinal helminth species of *R. rattus, R. norvegicus*, and *M. musculus*. Although some of these parasites reportedly infect humans, the low prevalence and abundance of these parasites suggest that they are of low concern for public health. The presence of gravid females of native parasites in invasive rodents supports the spillback hypothesis, but more studies are needed to test this hypothesis. Coinfection and correlated abundances are not frequent among helminth communities of rodents. The host's sex was the factor that is most frequently associated with parasitism, notably with female sex-biased parasitism observed in all cases.

## Data Availability Statement

The original contributions presented in the study are included in the article/Supplementary Material, further inquiries can be directed to the corresponding author.

## Ethics Statement

The animal study was reviewed and approved by Comité de Bioética of the Facultad de Ciencias Veterinarias.

## Author Contributions

CG-T, FF-S, AG-O, and CL-A examined the sample and mounted and identified parasites. JB-V, JS-R, BB-G, FL-C, HE-R, MS, AH, LM, and CL-A caught and processed the rodents. LM and CL-A designed the study and analyzed the data. CL-A wrote the first manuscript draft. All authors revised and expanded upon the original draft and approved the submitted manuscript.

## Funding

This study was funded by the Fondo Nacional de Desarrollo Científico y Tecnológico (ANID/FONDECYT Grant Nos. 11170294 and 11150875) and the Vicerrectoría de Investigación y Desarrollo of the Universidad de Concepción (Grant No. 220.113.099).

## Conflict of Interest

The authors declare that the research was conducted in the absence of any commercial or financial relationships that could be construed as a potential conflict of interest.

## Publisher's Note

All claims expressed in this article are solely those of the authors and do not necessarily represent those of their affiliated organizations, or those of the publisher, the editors and the reviewers. Any product that may be evaluated in this article, or claim that may be made by its manufacturer, is not guaranteed or endorsed by the publisher.
