# Supplementary material for: Patterns of Gastrointestinal Helminth Infections in Rattus rattus, Rattus norvegicus, and Mus musculus in Chile
Source: Front Vet Sci. 2022 Jun 28;9:929208. doi: 10.3389/fvets.2022.929208 (PMC9277659; doi:10.3389/fvets.2022.929208)
Supplement: Supplementary file 3 [file Data_Sheet_1.docx]

Supplementary Material

# Table S2: Results of null model co-occurrence species by component communities with more than 3 helminth species with two of them presenting prevalence higher than 20%.

| Localities | Host species (n) | C-score index | | | P-value | |
| --- | --- | --- | --- | --- | --- | --- |
|  |  | Observed | Mean of simulated indices | Variance of simulated indices | observed ≤ expected | observed ≥ expected |
| Maipu 2 | *R. norvegicus* (12) | 3,857 | 3,659 | 0.055 |  | 0.200 |
| Pinto | *R. rattus* (14) | 2,821 | 2,980 | 0.030 | 0.217 |  |
| La Pintana | *M. musculus* (18) | 10,333 | 10,864 | 0.316 | 0.213 |  |

# Table S3: Pairwise Spearman correlation tests of abundance of helminths by species and study unit.

| **Host** | **Locality** | **Parasite species** | **Parasite species** | **Spearman *ρ*** | **P-value** |
| --- | --- | --- | --- | --- | --- |
| *Rattus rattus* | Castro | *Heterakis spumosa* | *Syphacia muris* | 0.47 | 0.89 |
| *Rattus rattus* | Carahue | *Heterakis spumosa* | *Syphacia muris* | 0.45 | 0.04 |
| *Rattus rattus* | Carahue | *Heterakis spumosa* | *Nippostrongylus brasiliensis* | 0.6 | 0.004 |
| *Rattus rattus* | Carahue | *Physaloptera* sp. | *Heterakis spumosa* | 0.03 | 0.9 |
| *Rattus rattus* | Carahue | *Physaloptera* sp. | *Syphacia muris* | 0.11 | 0.64 |
| *Rattus rattus* | Carahue | *Physaloptera* sp. | *Nippostrongylus brasiliensis* | 0.35 | 0.12 |
| *Rattus rattus* | Pinto | *Heterakis spumosa* | *Nippostrongylus brasiliensis* | 0.63 | 0.017 |
| *Rattus rattus* | Pinto | *Heterakis spumosa* | *Physaloptera* sp. | 0.03 | 0.9 |
| *Rattus rattus* | Pinto | *Heterakis spumosa* | *Pterygodermatites* sp. | -0.08 | 0.79 |
| *Rattus rattus* | Pinto | *Heterakis spumosa* | *Hymenolepis diminuta* | 0.17 | 0.57 |
| *Rattus rattus* | Pinto | *Heterakis spumosa* | *Protospirura* sp. | -0.15 | 0.62 |
| *Rattus rattus* | Pinto | *Nippostrongylus brasiliensis* | *Physaloptera* sp. | -0.17 | 0.56 |
| *Rattus rattus* | Pinto | *Nippostrongylus brasiliensis* | *Pterygodermatites sp.* | -0.25 | 0.38 |
| *Rattus rattus* | Pinto | *Nippostrongylus brasiliensis* | *Hymenolepis diminuta* | 0.08 | 0.79 |
| *Rattus rattus* | Pinto | *Nippostrongylus brasiliensis* | *Protospirura* sp*.* | -0.25 | 0.38 |
| *Rattus rattus* | Pinto | *Physaloptera* sp*.* | *Pterygodermatites* sp*.* | -0.11 | 0.7 |
| *Rattus rattus* | Pinto | *Physaloptera* sp*.* | *Hymenolepis diminuta* | -0.14 | 0.62 |
| *Rattus rattus* | Pinto | *Physaloptera* sp*.* | *Protospirura* sp*.* | 0.73 | 0.003 |
| *Rattus rattus* | Pinto | *Pteryogodermatites* sp. | *Hymenolepis diminuta* | -0.21 | 0.47 |
| *Rattus rattus* | Pinto | *Pteryogodermatites sp.* | *Protospirura* sp*.* | -0.17 | 0.57 |
| *Rattus rattus* | Pinto | *Hymenolepis diminuta* | *Protospirura* sp*.* | 0.18 | 0.54 |
| *Rattus norvegicus* | Maipú 1 | *Heterakis spumosa* | *Syphacia muris* | -0.48 | 0.11 |
| *Rattus norvegicus* | Maipú 1 | *Heterakis spumosa* | *Nippostrongylus brasiliensis* | -0.03 | 0.93 |
| *Rattus norvegicus* | Maipú 1 | *Heterakis spumosa* | *Physaloptera* sp*.* | 0.16 | 0.62 |
| *Rattus norvegicus* | Maipú 1 | *Heterakis spumosa* | *Pterygodermatites* sp*.* | 0.48 | 0.11 |
| *Rattus norvegicus* | Maipú 1 | *Heterakis spumosa* | *Hymenolepis diminuta* | -0.1 | 0.75 |
| *Rattus norvegicus* | Maipú 1 | *Heterakis spumosa* | Capillariidae | -0.1 | 0.75 |
| *Rattus norvegicus* | Maipú 1 | *Syphacia muris* | *Nippostrongylus brasiliensis* | -0.28 | 0.37 |
| *Rattus norvegicus* | Maipú 1 | *Syphacia muris* | *Physaloptera* sp*.* | -0.21 | 0.51 |
| *Rattus norvegicus* | Maipú 1 | *Syphacia muris* | *Pterygodermatites* sp*.* | -0.09 | 0.78 |
| *Rattus norvegicus* | Maipú 1 | *Syphacia muris* | *Hymenolepis diminuta* | 0.46 | 0.13 |
| *Rattus norvegicus* | Maipú 1 | *Syphacia muris* | Capillariidae | -0.17 | 0.59 |
| *Rattus norvegicus* | Maipú 1 | *Nippostrongylus brasiliensis* | *Physaloptera* sp*.* | -0.25 | 0.43 |
| *Rattus norvegicus* | Maipú 1 | *Nippostrongylus brasiliensis* | *Pterygodermatites* sp*.* | -0.28 | 0.37 |
| *Rattus norvegicus* | Maipú 1 | *Nippostrongylus brasiliensis* | *Hymenolepis diminuta* | 0.21 | 0.51 |
| *Rattus norvegicus* | Maipú 1 | *Nippostrongylus brasiliensis* | Capillariidae | 0.25 | 0.43 |
| *Rattus norvegicus* | Maipú 1 | *Physaloptera* sp*.* | *Pterygodermatites* sp*.* | 0.37 | 0.24 |
| *Rattus norvegicus* | Maipú 1 | *Physaloptera* sp*.* | *Hymenolepis diminuta* | -0.06 | 0.84 |
| *Rattus norvegicus* | Maipú 1 | *Physaloptera* sp*.* | Capillariidae | 0.38 | 0.22 |
| *Rattus norvegicus* | Maipú 1 | *Pterygodermatites* sp*.* | *Hymenolepis diminuta* | -0.17 | 0.59 |
| *Rattus norvegicus* | Maipú 1 | *Pterygodermatites* sp*.* | Capillariidae | -0.17 | 0.59 |
| *Rattus norvegicus* | Maipú 1 | *Hymenolepis diminuta* | Capillariidae | 0.07 | 0.82 |
| *Mus musculus* | Maipú 1 | *Syphacia obvelata* | *Physaloptera* sp. | 0.37 | 0.2 |

# Table S4: Parameters of the logistic regression model “Presence of *Heterakis spumosa* = sex + latitude + body condition”. The basal level of sex, 'female', is given in parenthesis, as well as the parameter (odds ratio) is given for the dummy variable 'male'.

| Log likelihood=-45.641 | | | P-value=0.001 | |
| --- | --- | --- | --- | --- |
| Variable | Odds ratio | Standard Error | P-value | Confidence interval |
| Sex (Female) |  |  |  |  |
| Male | 0.37 | 0.2 | 0.07 | 0.13 – 1.09 |
| Latitude | 1.21 | 0.1 | 0.03 | 1.02 – 1.43 |
| Body condition | 0.67 | 0.15 | 0.07 | 0.44 – 1.03 |
| P-value of Goodness-of-fit test: 0.47 | | | | |

# Table S5: Parameters of the logistic regression model “Presence of *Nippostrongylus brasiliensis* = sex”. The basal level of sex, 'female', is given in parenthesis, as well as the parameter (odds ratio) is given for the dummy variable 'male'.

| Log likelihood=-38.88 | | | P-value=0.007 | |
| --- | --- | --- | --- | --- |
| Variable | Odds ratio | Standard Error | P-value | Confidence interval |
| Sex (Female) |  |  |  |  |
| Male | 0.16 | 0.13 | 0.02 | 0.33 – 0.75 |
| P-value of Goodness-of-fit test: >0.99 | | | | |

# Table S6: Parameters of the logistic regression model “Presence of *Physaloptera* = body condition”.

| Log likelihood=-24.98 | | | P-value=0.02 | |
| --- | --- | --- | --- | --- |
| Variable | Odds ratio | Standard Error | P-value | Confidence interval |
| Body condition | 0.41 | 0.2 | 0.07 | 0.16 – 1.08 |
| P-value of Goodness-of-fit test: >0.99 | | | | |

# Table S7: Parameters of the logistic regression model “Presence of *Hymenolepis diminuta* = sex + latitude”. The basal category of sex, 'female', is given in parenthesis, as well as the parameter (odds ratio) is given for the dummy variable 'male'.

| Log likelihood=-31.149 | | | P-value=0.045 | |
| --- | --- | --- | --- | --- |
| Variable | Odds ratio | Standard Error | P-value | Confidence interval |
| Sex (Female) |  |  |  |  |
| Male | 0.45 | 0.34 | 0.29 | 0.10 – 1.98 |
| Latitude | 0.67 | 0.15 | 0.17 | 0.43 – 1.03 |
| P-value of Goodness-of-fit test: 0.41 | | | | |

# Table S8: Parameters of the negative binomial regression model “Abundance of *Heterakis spumosa* = latitude + body condition”.

| Log likelihood=-115.978 | | | P-value<0.001 | |
| --- | --- | --- | --- | --- |
| Variable | Coefficient | Standard Error | P-value | Confidence interval |
| Latitude | 0.47 | 0.12 | <0.001 | 0.23 – 0.7 |
| Body condition | -1.2 | 0.36 | 0.001 | -1.89 – -0.5 |

# Table S9: Parameters of the negative binomial regression model “Abundance of *Syphacia obvelata* = body condition”.

| Log likelihood=-56.751 | | | P-value=0.001 | |
| --- | --- | --- | --- | --- |
| Variable | Coefficient | Standard Error | P-value | Confidence interval |
| Body condition | 1.83 | 0.91 | 0.04 | 0.047 – 3.61 |

# Table S10: Parameters of the negative binomial regression model “Abundance of *Trichuris muris* = sex”. The basal level of sex, 'female', is given in parenthesis, as well as the parameter (coefficient) is given for the dummy variable ‘male’.

| Log likelihood=-49.08 | | | P-value=0.04 | |
| --- | --- | --- | --- | --- |
| Variable | Coefficient | Standard Error | P-value | Confidence interval |
| Sex (Female) |  |  |  |  |
| Male | -3.88 | 1.49 | 0.01 | -6.81 – -0.96 |

# Table S11: Parameters of the negative binomial regression model “Abundance of *Nippostrongylus brasiliensis* = sex”. The basal level of sex, 'female', is given in parenthesis, as well as the parameter (coefficient) is given for the dummy variable 'male'.

| Log likelihood=-90.873 | | | P-value<0.001 | |
| --- | --- | --- | --- | --- |
| Variable | Coefficient | Standard Error | P-value | Confidence interval |
| Sex (Female) |  |  |  |  |
| Male | -5.18 | 1.1 | <0.001 | -7.33 – -3.03 |

# Table S12: Parameters of the negative binomial regression model “Abundance of *Physaloptera* sp. = sex + latitude”. The basal category of sex, 'female', is given in parenthesis, as well as the parameter (coefficient) is given for the dummy variable 'male'.

| Log likelihood=-68.315 | | | P-value=0.028 | |
| --- | --- | --- | --- | --- |
| Variable | Coefficient | Standard Error | P-value | Confidence interval |
| Sex (Female) |  |  |  |  |
| Male | -1.36 | 0.72 | 0.06 | -2.77 – -0.06 |
| Latitude | -0.32 | 0.15 | 0.04 | -0.62 – -0.02 |

# Table S13: Parameters of the negative binomial regression model “Abundance of Capillariidae = latitude”.

| Log likelihood=-34.07 | | | P-value=0.02 | |
| --- | --- | --- | --- | --- |
| Variable | Coefficient | Standard Error | P-value | Confidence interval |
| Latitude | -1.62 | 0.65 | 0.01 | -2.9 – -0.34 |
